# The Selective Action of Toxins on Nervous Tissues

**Published:** 1901-02-02

**Authors:** 


### HOSPITAL CLINICS AND MEDICAL PROGRESS.

### THE SELECTIVE ACTION OF TOXINS ON NERVOUS TISSUES.

According to modern theories many of the distinctive pathological conditions met with in different diseases, as well as the symptoms they produce, are due to the selective interaction between the different poisons which are present in these maladies and the various tissues. Discussing this subject in regard particularly to the nervous system, Dr. Mott[1] says that what is spoken of as the "new neurology" does not involve merely an acceptance of the neuron theory, but a recognition of the fact that every cell possesses a bio-chemical sensitiveness to its lymph environment, termed chemiotaxis. The essential cause then of a class of diseases of the nervous system which includes functional disorders and primary degenerations, is the failure of the neuron, under the influence of an altered environment or owing to inherent vital prostration, to carry on the processes of assimilation and dissimilation, which are essential for the well-being of every cell; and the alteration of the environment consists in many cases of the presence of some form of poison.

One great peculiarity about these primary intoxications and degenerations is that, as a rule, they are symmetrical, and are related to the functions of groups, systems, and communities of neurons. They are occasioned directly or indirectly by some general toxic condition of the blood or lymph, but although the toxic substance circulating in the blood must come in contact with all the neurons equally, yet we find that in a great number of instances particular groups of neurons, subserving special functions, are especially, and indeed not infrequently, solely affected by particular poisons. It is this selective action of certain poisons which impresses on our minds a clinical picture that enables us to recognise the cause of the disease by the character of the symptoms. As illustrations of this one may cite tetanus and rabies and the effects of the chemical poison strychnine. Whatever the individual temperament of the person attacked tetanus and strychnine have a specific action upon the spinal motor neurons, and act upon all individuals in precisely the same manner. There are, however, a great number of diseases of the nervous system in which toxic agents are not the sole cause, in which poisons circulating in the blood act rather as contributory, predisposing, or exciting causes in persons with a neuropathic or psychopathic heredity, or in persons who have subjected their nervous systems to excessive functional activity or stress. Thus we have in a majority of cases three factors in operation which together form a vicious circle—toxæmia, stress, and hereditary neurosis or psychosis. Obviously, whatever sociologists may in time do for the race, the only factors which can be removed in the case of the individual are the first two of those mentioned, namely, toxæmia and stress.

The poisons by which disease of the nervous system may be produced, either directly by their selective action on certain groups of neurons or indirectly by their injurious effect on neurons whose excitability has been lowered by their having been exposed to stress or from their being hereditarily feeble, are of considerable variety. They may either be introduced into the body from without or they may be produced within—be either exogenetic or autogenetic. In either case it is the toxic state of the blood or lymph which causes the morbid nervous phenomena, but the symptoms which arise depend upon conditions of either increased excitability or depressed excitability of the nervous elements which are selected by the poison. Which nervous elements are picked out, and the direction in which they are affected, depends in the one case (*a*) upon the selective influence of the poison on particular nervous structures, and this is dependent upon the chemical properties of the poison itself. In this case, therefore, the poison has a specific effect. In the other case (*b*) however, the poison selects particular structures in the nervous system owing to an inherited or acquired low potential of these nervous elements, or an instability of their protoplasm.

Of the extrinsic causes of nervous and mental disease the abuse of alcohol is the most widespread and potent, for at least 20 per cent. of the inmates of the London county asylums are suffering directly from the effects of the abuse of alcohol; but the list of the exogenetic poisons is a long one. The poisons formed within the body (autogenetic) by which the nervous system may be affected, may be produced (1) by the perverted functions of the organs or tissues, (2) by the action of micro-organisms upon the living fluids and tissues of the body. Even certain normal constituents of the blood may accumulate in excess, and toxic environment of the neuron may supervene, as for example in uræmia and Graves's disease. In the process of digestion a number of toxic albumoses and other substances are produced which, although normal enough in their proper place, are abnormal in the blood. By the vital action of the living epithelium of the alimentary canal and by the bio-chemical processes occurring in the liver, these substances are usually kept out of the systemic circulation. If, however, these structures are prevented by any cause from performing their normal function, toxic conditions of the blood may arise and be a source of morbid nervous phenomena.

Again, from defective metabolism abnormal substances may be found in the blood, and may be associated with various nervous disturbances. The very term "melancholia," signifying as it does "black bile," indicates the importance which has long been attached to the liver as an organ, the derangement of which causes nervous depression. The delirium, coma, and other symptoms occurring in acute yellow atrophy of the liver demonstrate the important part played by this organ in maintaining constant the normal quality or the blood. Again in diabetic coma we have an example of auto-intoxication, while in certain grave anæmias the degenerative changes found in the spinal cord are due not so much to the defect in the red corpuscles as to some toxic agent absorbed with the portal blood which not only leads to hæmolysis, but acts as a poison upon those nervous structures which are found in a state of degeneration.

A certain number of cases of puerperal insanity are due to septic blood-poisoning in psychopathic or neuropathic individuals, and the nervous phenomena connected with other infections are familiar to all. The best examples of selective actions of microbial toxins are afforded by rabies and tetanus, but the toxin of diphtheria may also be cited, and the tendency of syphilis to select certain areas of the nervous system is well known. We have then to recognise that poisons introduced into the blood stream, and thus into the lymph by which the tissues are fed, do not merely cause a generalised interference with the functions of the nervous system, but have a selective action, so that each poison tends to fall with especial force upon certain special groups or families of neurons; and that this selection is in some cases specific to the individual poison, while in some it is due rather to the tendency of the poison to fall upon such weakly neurons as have been enfeebled by stress or by hereditary taint. To this we would add that the action of these poisons appears sometimes to be limited to disturbance of function, while it sometimes leads to definite changes such as the neuritis; and further that it is very probable that even where the effect of the poison is so slight as not to give rise to obvious symptoms at the time, the final effect may be that degenerative changes may occur in the areas of nervous tissue which had been picked out by the poison at a far earlier date than would otherwise have been the case. In this way, perhaps, we may explain the relation between certain system degenerations of the nervous system, such as those producing ataxy and general paralysis, and some long-forgotten attack of syphilis.

[1] Lancet, Jan. 26.

